# Determinants of Dental Service Use Based on the Andersen Model: A Study Protocol for a Systematic Review

**DOI:** 10.3390/healthcare8030333

**Published:** 2020-09-10

**Authors:** André Hajek, Benedikt Kretzler, Hans-Helmut König

**Affiliations:** Department of Health Economics and Health Services Research, University Medical Center Hamburg-Eppendorf, 20246 Hamburg, Germany; b.kretzler.ext@uke.de (B.K.); h.koenig@uke.de (H.-H.K.)

**Keywords:** dental service, dental visits, dental care use, dental care utilization, oral health services, Andersen model

## Abstract

Introduction: Drawing on the Andersen model, there is a large body of evidence examining the determinants of health care use, such as doctor visits or hospitalization. However, far less is known about the determinants of dental service use, explicitly using the Andersen model. Consequently, the aim of this systematic review is to summarize and critically analyze evidence from observational studies that examine the determinants of dental service use based on the Andersen model. Methods and analysis: The following electronic databases will be searched: PubMed, PsycInfo, and CINAHL. Our main inclusion criterion is: observational studies (cross-sectional and longitudinal) investigating the determinants of dental service use based on the Andersen model. Disease-specific samples will be excluded. Data extraction will concentrate on methods (such as measurement of dental service use), sample characteristics (such as age and gender) and key results. The study quality will be assessed using an appropriate tool. Three steps (selecting the studies, extracting the data and assessment of study conducted) will be performed by two reviewers. The findings will be displayed using figures, summary tables, narrative summaries and meta-analysis (if studies are deemed similar enough and of appropriate quality).

## 1. Introduction

Health care use (HCU) particularly includes hospitalization and outpatient physician visits, such as general practitioner or specialist visits (e.g., neurologists or ophthalmologists). Dental visits are also an important component of health care use [1,2]. Given increased use of health care services is commonly associated with significant economic cost, knowledge about the factors associated with health care use is important. Specifically, this knowledge may help to manage dental service use, and may also help to avoid over- and underuse, as well as misuse of services [3].

Frequent dental visits is also associated with overtreatment [4], and increased negative emotions [5]. On the other hand, postponed dental visits due to financial restrictions can lead to tooth loss [6].

Various studies have examined the determinants of general health care use, e.g., doctor visits or hospital stays [7], drawing on the Andersen model [8]. This model incorporates both contextual and individual determinants of HCU; or, as stated by Andersen [9]: “[it] divides the major components of contextual characteristics in the same way as individual characteristics have traditionally been divided—those that predispose …, enable …, or suggest need for individual use of health services” (p. 652). Thus, it distinguishes between various determinants associated with health care use, namely predisposing characteristics (sex or age), enabling resources (income or perceived access to HCU) and need factors (self-rated health or chronic illness).

More precisely, individual predisposing factors include “biological factors” such as age and sex, or social factors such as education and social relationships. Moreover, contextual predisposing factors include, among other things, social composition of communities or cultural norms.

Enabling resources include organizational and financial factors which may drive HCU. Individual financing factors include wealth and income (to pay for regular health services or out-of-pocket payments). Organizational factors cover, for example, travel time, waiting time for health care, transportation. Moreover, contextual factors include, among other things, physician and hospital density, rate of health insurance coverage, and the relative price of services and goods.

Individual need factors can be divided into perceived need (e.g., self-rated health) and evaluated need (such as physician diagnosed illnesses). Contextual need factors cover environmental need characteristics (such as crime-related injury or traffic) and population health indices (such as indicators of disability or mortality).

It is worth noting that the Andersen model can be considered as a very important, widely acknowledged and widely used model to study the determinants of HCU [8]. Moreover, there are also some studies that have examined the determinants of dental service use, based on the Andersen model ([10,11,12]). These studies showed, among other things, that—depending on the healthcare system—all components of the Andersen model (predisposing characteristics, enabling resources and need factors) are important determinants of dental service use. It is often the goal of health systems to ensure equitable access to dental service use, by seeking to promote need factors as the driver of dental service use (i.e., by reducing the influence of predisposing and particularly enabling factors) [13]. Otherwise, this may indicate an over- or underuse of dental services. However, no systematic review has summarized the evidence on the determinants of dental service use based on this model. Therefore, the aim of this systematic review was to fill this gap. This means that we will synthesize observational studies examining the determinants of dental service use drawing on the Andersen model. This knowledge may assist in managing dental service use.

## 2. Methods and Analysis

This review’s methods draw on the Preferred Reporting Items for Systematic Reviews and Meta-Analysis Protocols (PRISMA-P) guidelines [14]. Furthermore, we have submitted this review to the International Prospective Register of Systematic Reviews (PROSPERO, registration number: CRD42020193094). In the final systematic review, we will include a PRISMA checklist.

### 2.1. Eligibility Criteria

Prior to determining final eligibility criteria, we will perform a pretest. To this end, we will screen 50 titles/abstracts. The criteria will be adapted if needed following this pretest.

#### 2.1.1. Inclusion Criteria

Inclusion criteria are:

- cross-sectional and longitudinal observational studies investigating the determinants of dental service use;
- studies based on the Andersen model;
- assessment of key variables with appropriate tools (e.g., self-reported frequency of dental visits in the last 3 months; claims data) which means that important variables are assessed in a valid manner;
- studies in English or German language, published in a peer-reviewed, scientific journal.

#### 2.1.2. Exclusion Criteria

Exclusion criteria are:

- studies solely investigating samples with a specific disorder (e.g., individuals with cognitive disorders);
- assessment of key variables not appropriate (e.g., using an unclear period for the frequency of dental visits).

PubMed, PsycInfo, and CINAHL will be searched by two authors (AH, BK). Predefined terms will be applied. In Table A1 (Appendix A), the search strategy for PubMed is displayed. No restrictions will be applied with regard to the time and location of the publication. Two reviewers will manually search the reference lists of studies that meet our final inclusion criteria.

### 2.2. Data Management

The data will be imported into Endnote X7 (Clarivate Analytics, Philadelphia, PA, USA) and a meta-analysis (if possible, which means that studies must be similar and of certain quality) will be performed using Stata 16.0 (StataCorp, College Station, TX, USA).

### 2.3. Study Selection Process

Two reviewers (AH, BK) will assess titles/abstracts against the eligibility criteria. Following this, both reviewers will review the full texts. If necessary, discussions will be held to ensure that consensus is reached (if required, a third party (HHK) will be included).

### 2.4. Data Collection Process and Data Items

One reviewer will perform data extraction. The second reviewer will cross-check it. Thus, data extraction will be conducted by two reviewers (AH, BK). Discrepancies, if identified, will be discussed by two reviewers (AH, BK). A third party will be included in discussions (HHK), if discrepancies are not resolved. If clarification on details of included studies is needed, the study authors will be contacted for more information.

We will focus on extracting data related to study design, explanatory variables (in terms of predisposing characteristics, enabling resources and need factors), assessment of dental service use, characteristics of the sample (including country of origin), statistical approach used and the main results of the study.

### 2.5. Assessment of Study Quality/Risk of Bias

We will use an appropriate tool (i.e., HCU tool developed by Hohls et al. [15]) to assess study quality. It has also been applied in recent studies [15,16]. We will apply this specific tool for reasons of clarity, applicability and succinctness. This HCU tool [15] include important criteria consisting of six groups (arranged by their topic): “scope” (e.g., “study objective” or “inclusion and exclusion criteria”), “general HCU” (e.g., “HCU description” or “comparison group or disorder-specific HCU”), “study design and analysis” (e.g., “missing data” or “sensitivity analysis”), “presentation of results” (e.g., “sample size” or “demographics”), “discussion” (e.g., “results discussed regarding generalizability” or “conclusion supported by data”), and “general” (e.g., “conflict of interest stated”). More details are given by Hohls et al. [15] in their Table A1.

Two reviewers (AH, BK) will assess the study quality. Difference in opinion will be resolved through discussion (if necessary, a third party (HHK) will be included).

### 2.6. Data Synthesis

The findings will be presented using figures, summary tables, narrative summaries and a meta-analysis (if studies are deemed similar enough and of appropriate quality). We will display the study selection process using a flow chart in the systematic review.

We plan to categorize the key results according to the Andersen model (distinguishing between predisposing characteristics, enabling resources and need factors) and between curative and preventive dental visits (if possible).

### 2.7. Patient and Public Involvement Statement

The present review protocol did not involve individual patients or public agencies.

## 3. Discussion

Some recent empirical studies (e.g., [10,11,12]) have emphasized the importance of all components of the Andersen model for dental service use. This may point to some over- or underuse of dental services. However, to date, there is a lack of systematic reviews systematically summarizing studies that have investigated the determinants of dental service use, drawing on the Andersen model. Our aim was to fill this gap. In addition, we will assess the quality of studies on this topic.

Strengths and Limitations

To the best of our knowledge, this is the first systematic review that summarizes the determinants of dental service use based on the Andersen model. A main strength is that steps such as study selection, data extraction and assessment of study quality will be conducted by two reviewers. If data permit, a strength will be that a meta-analysis will be performed. The inclusion of peer-reviewed articles ensures a certain quality of the studies. This could be considered as a strength. However, a possible limitation is that other important articles are excluded (e.g., from grey literature). While we included three main electronic databases, such as PubMed, for our search, the possibility cannot be dismissed that other databases could identify more important articles. This is a potential limitation of this study. A further limitation is the focus on studies in German or English language.

## 4. Conclusions

Our review may identify potential gaps in knowledge, such as the general lack of longitudinal studies. However, longitudinal studies are required to derive insights into potential causal mechanisms, and to provide consistent estimates [17]. Furthermore, our review may reveal that some studies do not differentiate between curative and preventive dental service use. If our systematic review determines a link between enabling resources and actual dental service use, our study may contribute to the debate on inequality in dental service use [18]. However, if most of the studies included in our review demonstrate a link between need factors and dental service use, this may point to an adequate use of dental services. The findings of this review may be influenced by the health care system of the country where the study takes place (e.g., private vs. publicly funded healthcare system).

## 5. Ethics and Dissemination

No primary data will be collected. Therefore, approval by an ethics committee is not required. We plan to publish our findings in a peer-reviewed journal.

## Appendix A

**Table A1:** Search strategy (PubMed).

#	Search Term
#1	Dental serv *
#2	Dental visit *
#3	Dental care u *
#4	Oral health serv *
#5	Dentist
#6	#1 OR #2 OR #3 OR #4 OR #5
#7	Andersen model
#8	Andersen’s behavioral model of healthserv *
#9	Andersen and Newman behavioralmodel of health serv *
#10	#7 OR #8 OR #9
#11	#6 AND #10

Note: The asterisk (*) is a truncation symbol. The number sign (#) refers to the search order.
